# Biomarkers for Immune Checkpoint Inhibitor Response in NSCLC: Current Developments and Applicability

**DOI:** 10.3390/ijms241511887

**Published:** 2023-07-25

**Authors:** Katiane Tostes, Aléxia Polo Siqueira, Rui Manuel Reis, Leticia Ferro Leal, Lidia Maria Rebolho Batista Arantes

**Affiliations:** 1 Molecular Oncology Research Center, Barretos Cancer Hospital, Barretos 14784-400, São Paulo, Brazil; katianetostes@hotmail.com (K.T.);; 2 Life and Health Sciences Research Institute (ICVS), Medical School, University of Minho, 4710-057 Braga, Portugal; 3 ICVS/3B’s-PT Government Associate Laboratory, 4806-909 Guimarães, Portugal; 4 Barretos School of Health Sciences, Dr. Paulo Prata-FACISB, Barretos 14785-002, São Paulo, Brazil

**Keywords:** immunotherapy, immune checkpoint inhibitors, non-small cell lung cancer, PD-L1, predictive biomarkers

## Abstract

Lung cancer has the highest mortality rate among all cancer types, resulting in over 1.8 million deaths annually. Immunotherapy utilizing immune checkpoint inhibitors (ICIs) has revolutionized the treatment of non-small cell lung cancer (NSCLC). ICIs, predominantly monoclonal antibodies, modulate co-stimulatory and co-inhibitory signals crucial for maintaining immune tolerance. Despite significant therapeutic advancements in NSCLC, patients still face challenges such as disease progression, recurrence, and high mortality rates. Therefore, there is a need for predictive biomarkers that can guide lung cancer treatment strategies. Currently, programmed death-ligand 1 (PD-L1) expression is the only established biomarker for predicting ICI response. However, its accuracy and robustness are not consistently reliable. This review provides an overview of potential biomarkers currently under development or in the validation stage that hold promise in improving the classification of responders and non-responders to ICI therapy in the near future.

## 1. Introduction

### 1.1. Non-Small Cell Lung Cancer

The World Health Organization (WHO) estimated in 2020 an incidence of about 2.2 million new lung cancer cases and 1.8 million deaths due to lung cancer. Histologically, lung cancer can be classified into two primary groups: small cell lung cancer (SCLC), which accounts for 15% of cases, and non-small cell lung cancer (NSCLC), which accounts for 85% of cases. Within the NSCLC category, three main subtypes exist: adenocarcinoma (ADC), squamous cell carcinoma (SCC), and large cell carcinoma (LCC) [1].

ADC is the most common subtype of NSCLC, accounting for approximately 40% of cases. ADC arises from epithelial cells in the peripheral region of the lung and is not exclusively associated with smoking. Nevertheless, it is noteworthy that ADC cases are indeed associated with smoking habits [1,2]. SCC is the second-most prevalent subtype, representing 25–30% of all lung cancer cases. SCC is characterized by a malignant tumor derived from keratinized epithelial cells and is strongly associated with tobacco use. Typically, it arises from the central or lobar bronchus [1,3]. The third subtype, LCC, is frequently diagnosed in current or former smokers and accounts for 1 to 8% of cases [1,4]. LCC is characterized by poorly differentiated cells that lack the distinctive features of ADC and SCC. Additionally, a small proportion of poorly differentiated lung carcinomas are classified as unspecified or not otherwise specified (NOS). The pathological diagnosis of these poorly differentiated subtypes relies on exclusion. However, advancements in immunohistochemical techniques and specific biomarkers have overcome some of the limitations in distinguishing squamous carcinomas and adenocarcinomas [1,3].

### 1.2. Non-Small Cell Lung Cancer Treatment Strategies

NSCLC patients receive different treatment approaches based on disease stage. The majority of patients are diagnosed with metastases [5,6]. Among patients undergoing palliative chemotherapy (CT) for metastatic disease, the observed overall survival (OS) ranges from 8 to 18 months [7]. In this context, the continuous advances in precision medicine for tailored treatment contribute positively to survival outcomes [8].

Platinum-based CT has historically been the gold standard treatment for patients with advanced disease [9]. In 2002, the Eastern Cooperative Oncology Group (ECOG) phase III clinical trial data demonstrated no difference in OS between taxane or gemcitabine in platinum-based CT regimens [10]. A significant breakthrough occurred in 2006 when anti-VEGF therapy (bevacizumab) was approved for treating non-squamous carcinoma [11]. Subsequently, a pemetrexed and platinum combination, followed by pemetrexed consolidation, was approved, showing lower toxicity and improved OS outcomes compared to other platinum-based regimens for non-squamous NSCLC patients [12].

The major advance in metastatic NSCLC treatment has been the development of targeted therapies for driver mutations [13]. However, about 50% of NSCLC patients are eligible for targeted treatment [14]. Currently, tailored therapies focus on driver alterations in genes such as *EGFR*, *ALK*, *ROS1*, *NTRK*, *KRAS* (*p.G12C*), *BRAF*, *RET*, and *cMET* [15]. About 50–80% of advanced NSCLC (aNSCLC) patients with driver alterations in *EGFR*, *ALK*, *ROS1*, and *BRAF* present survival improvements after receiving targeted therapies [7]. In 2015, immune checkpoint inhibitors (ICI) treatment emerged as a new option for metastatic NSCLC patients [16].

## 2. Immunotherapy: Immune Checkpoint Inhibitors

The immune system can distinguish and attack antigen-carrying cells [17]. Cells modulate the immune response through immunological checkpoints, avoiding excessive inflammatory reactions or autoimmunity. Cancer immunotherapy relies on the immune system’s ability to recognize and eliminate tumor cells as potential threats. T lymphocytes express checkpoint proteins following antigen recognition, leading to immune cell proliferation. However, chronic activation of these receptors in the tumor microenvironment (TME) can induce T lymphocyte exhaustion, resulting in a dysfunctional immune response [18].

Exhausted T lymphocytes lose their effector functions and start to continuously express multiple inhibitory receptors [19]. While these events prevent immune cells from attacking normal cells, T lymphocyte exhaustion hampers the anti-tumor immune response [20]. Cytotoxic T-Lymphocyte Antigen-4 (CTLA-4) and the Programmed Cell Death Protein 1 (PD-1) are the most explored inhibitory receptors [21]. Additional inhibitory receptors such as V-domain Ig suppressor of T cell activation (VISTA) [22], lymphocyte activation gene 3 (LAG-3) [19], T cell immunoglobulin- and mucin-domain-containing 3 (TIM-3) [23], and T cell immunoglobulin and ITIM domain (TIGIT) [23] have also been investigated. 

PD-1 is an important immune checkpoint expressed in various immune cells such as macrophages, B lymphocytes, dendritic cells (DCs), monocytes, activated T cells specific to tumors, myeloid cells, and natural killer (NK) cells in the presence of chronic antigen exposure. On the other hand, PD-L1 expression is primarily detected in tumor cells, tumor-infiltrating cells, and antigen-presenting cells (APCs) in numerous cancer types [24]. The interaction between PD-1 on CD8^+^ T lymphocytes and its ligand PD-L1 triggers phosphorylation of the immunoreceptor tyrosine-based inhibitory motif (ITIM) and the immunoreceptor tyrosine-based switch motif (ITSM) [25]. 

This mechanism triggers a dephosphorylation cascade of T-cell receptor (TCR) molecules, promoting anergy and exhaustion in T lymphocytes [26]. However, tumor cells exploit PD-L1 overexpression as a means to evade immune surveillance [27], promoting tumor initiation, progression, and metastasis [28]. PD-1/PD-L1 interactions attenuate TCR signaling and reduce effector functions [27]. Therefore, inhibitory receptors prevent immune cells from launching attacks against self-tissues [19]. Nonetheless, lymphocyte depletion can negatively impact the effectiveness of immune antitumor responses [29]. Immune evasion is considered a hallmark of cancer in the context of carcinogenesis [30].

Activation of immune checkpoints is mediated through ligand–receptor interactions. Thus, molecules capable of blocking these interactions have been used to reactivate antitumor T-lymphocyte immunity [31]. Monoclonal antibodies (mAbs) have shown efficacy in multiple cancer types by modulating immune responses [32,33]. 

Initially, ICIs were approved for NSCLC patients who experienced disease progression after platinum-based first-line treatment. Clinical trials such as CheckMate 057 [34], CheckMate 017 [35], Keynote 010 [36], and OAK [37] demonstrated improved survival for patients treated with nivolumab, pembrolizumab, and atezolizumab, respectively, compared to docetaxel treatment. Furthermore, combination therapies involving pembrolizumab with platinum-based CT [38] or pembrolizumab alone [39] exhibited greater efficacy and progression-free survival (PFS) improvements compared to CT regimens. Subsequently, first-line pembrolizumab was approved for treatment-naïve aNSCLC expressing PD-L1 ≥ 50% [40]. In the last few years, ICIs, either alone or in combination with chemotherapy, have also been approved for treating advanced tumors without actionable alterations [36]. 

Immunotherapy has also been integrated into the treatment of early-stage and local aNSCLC. Durvalumab was approved as consolidation therapy after chemoradiotherapy for stage III NSCLC, resulting in a survival improvement of 18.4 months compared to chemoradiotherapy alone and placebo [33]. For resectable NSCLC, atezolizumab was recently approved as adjuvant treatment after platinum-based CT for patients expressing PD-L1 ≥ 1% [41], and nivolumab plus platinum-based CT was also approved as neoadjuvant treatment [32].

## 3. Biomarkers

While immunomodulatory agents have shown promising results in treating refractory solid tumors [42], not all patients exhibit satisfactory responses. Immunotoxicity is a common occurrence, underscoring the need for predictive biomarkers to identify patients who would benefit from immunotherapy [43]. Furthermore, the high cost of immunotherapies poses a barrier to access, limiting this treatment option to a privileged subset of patients in low–middle-income countries.

### 3.1. PD-L1 Expression

PD-L1 expression is the currently used biomarker for predicting the response and clinical efficacy of ICIs in NSCLC (Table 1) [44]. Positive PD-L1 expression is associated with better outcomes and clinical response rates than those for patients with negative PD-L1 negative expression [45], regardless of smoking habits [46]. PD-L1 expression levels can be measured using the Tumor Proportion Score (TPS) or Combined Positive Score (CPS), where TPS represents the percentage of tumor cells expressing PD-L1 and CPS accounts for PD-L1 presence in both tumor and inflammatory cells. Pro-inflammatory signals associated with JAK2 transduction [47] and IFN-γ expression [48] may increase PD-L1 expression [49].

In a phase I study, PD-L1 surface expression levels > 5% were associated with a response to nivolumab, while patients with PD-L1 expression < 5% did not respond well to treatment [50]. In the phase III Keynote 024 study, patients with aNSCLC and high levels of PD-L1 expression (≥50%) demonstrated improved outcomes when treated with pembrolizumab compared to the platinum-based CT group [39]. Although patients with advanced squamous cell NSCLC showed better responses to nivolumab than to docetaxel regardless of PD-L1 expression, even patients with negative PD-L1 expression experienced increased PFS after ICI treatment [35].

However, despite these findings, PD-L1 TPS alone is not sufficiently accurate or reliable in predicting the response to immunotherapy [51]. The technical limitations of immunohistochemistry (IHC) in the context of PD-L1 expression analysis include variations in sample quality, as the measurements are conducted on tumor biopsies that may not fully capture tumor heterogeneity. Additionally, diverse staining protocols and the establishment of appropriate cut-off points for interpreting PD-L1 expression pose further challenges [52]. In addition, the predictive capacity of IHC in determining the response to ICIs across various histologies, such as SCC, remains limited [34,35]. In this context, the combination of TPS and CPS is a more robust biomarker [53].

PD-L1 identification in circulating tumor cells (CTCs) tends to be higher than in tissues, potentially enhancing quantification efficiency. However, results regarding its correlation with treatment response remain controversial, with studies reporting absence of or worse-lasting response to ICIs with higher pretreatment PD-L1^+^ CTCs, while others indicate the positive prognostic value of PD-L1 expression in CTCs [54,55,56]. Some studies found no correlation between PD-L1 expression in tumor biopsies and CTCs [57].

Tumor cells that do not express PD-L1 may still be accompanied by immune cells expressing this ligand. Additionally, tumor-infiltrating immune cells in the tumor environment demonstrate increased levels of PD-L1 expression, and the administration of ICI leads to an enhanced influx of CD8^+^ T lymphocytes in the tumor region [58]. 

Plasma soluble PD-L1 (sPD-L1) levels have been investigated as an alternative to PD-L1 TPS. While certain studies have found no correlation between pre-treatment levels of soluble programmed death-ligand 1 (sPD-L1) and OS [59], other investigations have established a significant association between elevated pre-treatment sPD-L1 levels and unfavorable outcomes, including higher rates of treatment failure [60]. Furthermore, increased or stable levels of soluble PD-1 (sPD-1) after two cycles of nivolumab were linked to a better prognosis [61].

Genetic variations were also evaluated as possible biomarkers. PD-L1 polymorphisms were associated with the efficacy of ICIs, being correlated with OS improvement [62]. Patients carrying *HER2* exon 20 [63], *ERBB4* [64], *KRAS G12C* [65], *FGFR4* [66], *ARID 1A*, and *ARID 1B* [67] mutations showed ICIs benefit. However, patients harboring *KRAS G12V* [65], *EGFR* [68], or *ALK* [69] alterations tend to have poorer outcomes.

**Table 1:** PD-L1-expression-based biomarkers for ICIs response in NSCLC.

Biomarker	Method	Key Finding
PD-L1	IHC	Patients manifesting a pronounced PD-L1 expression profile exhibit greater efficacy to treatment with ICI [70]. PD-L1 protein levels in NSCLC unveil intra-tumoral heterogeneity and substantial inter assay variability or discordance [71]. The presence of a reservoir of PD-1-negative effector T lymphocytes establishes an immune-privileged microenvironment that exerts a beneficial influence on patient survival [49].
PD-L1 in CTCs	CellSearch system	CTCs number was correlated with baseline tumor size [56].
PD-L1 in CTCs	Immunoflorescence	CTCs exhibit a higher frequency of positive PD-L1 expression compared to tissue samples [54].
PD-L1 polymorphism	Genotyping assay	PD-L1 polymorphisms are correlated with favorable OS outcomes in patients with NSCLC treated with nivolumab [62].
sPD-L1	ELISA	High levels of sPD-L1, in patients undergoing treatment with Nivolumab, was correlated with a decrease in median PFS [61]. Decrease in plasma levels of sPD-L1 was significantly associated with tumor regression in patients treated with ICIs [59].

PD-L1—programmed cell death protein ligand 1; IHC—immunohistochemistry; ICI—immune checkpoint inhibitors; NSCLC—non-small cell lung cancer; PD-1—programmed cell death protein 1; CTCs—circulating tumor cells; OS—overall survival; sPD-L1—soluble programmed cell death protein ligand 1; ELISA—enzyme-linked immuno sorbent assay; PFS—progression-free survival.

### 3.2. Gene-Expression-Based Biomarkers

Gene-expression-based biomarkers have been successfully employed in the field of oncology for the clinical management of cancer patients (Table 2) [72]. In patients treated with anti-PD-1, the expression levels of *CSF1 R* and *HCST* positively correlated with PD-L1 levels and high infiltration of CD8^+^ T cells [73]. Nevertheless, other potential ICI response signatures have been described, including the expression levels of *MAP1A/1B/1S/4/6/7D1/7D3* in ADC and SCC, where high expression of these genes was associated with favorable response to immunotherapy [74]. In NSCLC, *KAT2B* expression displayed a positive correlation with the levels of infiltrating immune cells and mRNA expression of immune checkpoint genes. Conversely, tumor tissues exhibited downregulation of *KAT2B* expression, which was associated with ineffective response to ICI and unfavorable prognosis in patients with lung ADC [75]. 

A TCR co-expression signature has been identified as a valuable predictor of prognosis in NSCLC patients undergoing immunotherapy. Elevated expression levels of this specific gene-set are indicative of more favorable treatment responses [76]. Additionally, a cancer-specific immune score model has been developed to predict ICI response with satisfactory performance, achieving an area under the curve (AUC) of 0.68, indicating its potential for accurately assessing ICI response [77].

Moreover, the gene expression profile within the TME holds predictive value for pathologic complete response and disease progression in patients receiving combined neoadjuvant chemoimmunotherapy. Tumors showing favorable response to treatment exhibited elevated levels of *IFNG*, *GZMB*, *NKG7*, and M1 macrophages whereas tumors prone to relapse following surgery exhibited heightened expression of genes such as *AKT1*, *BST2*, *OAS3*, and *CD8B* [78].

To construct an immune gene score, pivotal immune cells, human leukocyte antigens (HLAs), and immune checkpoints were selected and the immune-related genes of those three aspects of the TME were combined to construct the score. The score derived from these three aspects demonstrated the ability to predict the response to ICIs, achieving an area under the curve (AUC) of 0.737 at the 20-month mark. Remarkably, within this signature, patients exhibiting a higher hypoxia score demonstrated a stronger association with immunotherapy efficacy. A predictive model integrating this immune score, tumor mutational burden (TMB), and long non-coding RNA expression exhibited promising predictive potential for effective immunotherapy response, achieving an AUC of 0.814 at 20 months [79].

Efforts are ongoing to identify gene-expression-based signatures that can accurately predict response to immunotherapy. However, current studies are in the preliminary stages and require further validation before their potential for clinical implementation can be fully assessed [80].

**Table 2:** Gene-expression-based biomarkers for ICIs response in NSCLC.

Biomarker	Method	Key Finding
*HNRNPA2B1*, *IGF2BP2*, *NSUN4*, *ALYREF*	Public datasets	ADC patients classified as high risk presented improved outcomes and were associated with ICI efficacy [80].
Immune-score	Public datasets	Classification based on the immune-score was correlated with improved outcomes in ICI-treated patients [77].
*KAT2B*	Public datasets	Low *KAT2B* is correlated with immune infiltration and high TMB and associated with unfavorable outcomes in ICI-treated ADC patients [75].
MAP1A/1B/1S/4/6/7D1 /7D3	Public datasets/RT-qPCR	Expression of the MAP gene-set is correlated with the immunophenoscore, a predictor of response of PD-1 blockers and CTLA-4 [74].
*TCR* co-expression	Public datasets	High *TCR* co-expression indicated better ICI response and improved OS and PFS [76].

ADC—adenocarcinoma; ICI—immune checkpoint inhibitor; TMB—tumor mutation burden; RT-qPCR—reverse transcriptase quantitative polymerase chain reaction; PD-1—programmed cell death protein 1; CTLA-4—cytotoxic T-lymphocyte-associated protein 4; TCR—T cell receptor; OS—overall survival; PFS—progression-free survival.

### 3.3. Tumor Mutation Burden

TMB refers to the presence of non-synonymous mutations in the coding regions of the tumor genome [81]. High TMB indicates a greater number of neoantigens, which can activate the T-lymphocyte response [82]. In NSCLC, the mutagenic effects of smoking can lead to higher TMB rates [83]. Several studies have correlated TMB rates with response to ICIs [82,84], suggesting that a TMB of ten or more mutations per megabase is associated with improved PFS (Table 3) [85]. Patients treated with pembrolizumab and high TMB rates have demonstrated durable clinical benefits [86]. Additionally, in ADC patients, a high burden of clonal neoantigens has been linked to better outcomes and ICI responses [87].

However, it is worth noting that less than 10% of nonsynonymous mutations result in immunogenicity [88]. Additionally, there is currently no established cut-off point for non-synonymous mutations that can reliably predict clinical benefit [89]. In colorectal cancer (CRC) patients, elevated TMB levels and microsatellite instability (MSI) are associated with a more favorable prognosis [90]. However, the prevalence of MSI-high (MSI-H) status in NSCLC patients is rare, limiting its usefulness as a biomarker in this patient population [91]. Despite these limitations, the combination of TMB with other biomarkers such as PD-L1 expression or the neutrophil-to-lymphocyte ratio (NLR) holds promise, as it enhances prediction capabilities [92].

### 3.4. Complete Blood Count

Absolute complete blood count (CBC) values have been evaluated as potential biomarkers for predicting the response to ICIs by analyzing patients’ medical records (Table 4) [93]. In NSCLC, the presence of local inflammation results in an immune infiltrate rich in neutrophils [94]. The increase in NLR [95,96,97], myeloid-to-lymphoid ratio (M:L), absolute-neutrophil-count-to-absolute-lymphocyte-count ratio (ANC:ALC) and absolute neutrophil counts (post-ANCs) is associated with lower PFS [98] and OS [99]. Conversely, low platelet-to-lymphocyte ratio (PLR) [96,100], monocyte-to-lymphocyte ratio (MLR) [96], and high rates of ALC [101], absolute eosinophil count (AEC) [102], and relative eosinophil count (REC) [103] are associated with better PFS.

Low levels of hemoglobin (HGB) [104], red blood cell (RBC) counts, and hematocrit (HCT), which reflect anemia, are associated with shorter OS. In this case, the ICI response can be identified by the combination of NLR and HGB [95]. The Lung Immune Prognostic Index (LIPI) score, which takes into account the derived neutrophil-to-lymphocyte ratio (dNLR) and lactate dehydrogenase level (LDH), is also associated with OS, with patients scoring zero demonstrating favorable outcomes [85]. However, studies have demonstrated that high levels of dNLR are present in patients who experience early failure with ICI [105].

Peripheral blood biomarkers, particularly those obtained from routine examinations, represent a significant advancement in clinical practice [106]. Nonetheless, further investigation is necessary to fully understand and validate these findings.

**Table 4:** Complete blood-count-based biomarkers, evaluated using the medical records of the patients, for ICIs response in NSCLC.

Biomarker	Key Finding
AEC	High AEC (≥130/μL) is correlated with improved PFS and OS [97,102].
ALC	High ALC (>0.93 × 109/L) is correlated with improved OS [101].
ANC	High ANC (>7.5 × 109/L) is correlated with shorter PFS and OS [97,98,99].
ANC:ALC	High ANC:ALC (≥5.9) is correlated with shorter PFS and OS [99].
dNLR	High dNLR (≥2.8 ± 0.2) is correlated with shorter PFS and OS, as well as early failure to ICIs [107].
HGB	High baseline HGB (≥110 g/L) is correlated with improved PFS and OS [95].
M:L	High M:L (≥11.3) is correlated with shorter OS [99].
MLR	High MLR (≥0.54) is correlated with shorter PFS and OS [96].
NLR	High NLR (≥4.0 ± 1.5) is correlated with shorter PFS and OS [96,97].
PLR	High PLR (≥183 ± 15) is correlated with shorter PFS and OS [96,100].

AEC—absolute eosinophil count; PFS—progression-free survival; OS—overall survival; ALC—absolute lymphocyte count; ANC—absolute neutrophil count; dNLR—derived neutrophil-to-lymphocyte ratio; ICI—immune checkpoint inhibitor; HGB—hemoglobin; M:L—myeloid-to-lymphoid ratio; MLR—monocyte-to-lymphocyte ratio; NLR—neutrophil-to-lymphocyte ratio; PLR—platelet-to-lymphocyte ratio.

### 3.5. Peripheral Blood Mononuclear Cells

Peripheral blood mononuclear cells (PBMCs) encompass various immune cell types, including monocytes, T cells, B cells, granulocytes, and natural killer cells (NK), and they play a crucial role in the initial immune defense against malignancies [108]. Regulatory T cells (Tregs) and myeloid-derived suppressor cells (MDSCs) are implicated in tumor growth and exert immunosuppressive functions in cancer patients [109].

In NSCLC patients treated with anti-PD-1 therapy, responders exhibit increased proliferation of T PD-1^+^CD8^+^cells with an effector phenotype (HLA-DR^+^ CD38^+^ BCL-2^low^) and elevated expression of CD28 (Table 5) [110]. High proliferation of PD-1^+^CD8^+^ T cells [111], PD-L1^−^ expressing CD14^+^ monocytes [112] and Forkhead Box P3 (FoxP3^+^) Treg cells [113] may also be associated with treatment response. However, an augmented frequency of TIM-3^+^ T lymphocytes, whether CD4^+^ or CD8^+^ T cells, negatively correlates to PFS [114]. Likewise, high levels of CCR9^+^ or CCR10^+^CD4^+^ T cells or CXCR4^+^CD8^+^ T cells negatively correlate with ICI treatment survival outcomes [115].

Nivolumab-treated NSCLC patients with a high central memory/effector T cell ratio demonstrate prolonged PFS and higher tumor PD-L1 expression [116]. Furthermore, an increase in exhausted cells (TIGIT^+^) and a decrease in memory effector CD8^+^ T cells (CCR7^−^ CD45RA^−^) are associated with disease progression [117].

The ratio of Tregs to Lox-1^+^ PMN-MDSCs (TMR) can be employed after the initial immunotherapy infusion and exhibits higher sensitivity in predicting negative treatment responses [109]. In contrast, high levels of granulocytic myeloid-derived suppressor cells (Gr-MDSC) are related to a positive response to treatment with ICIs [97]. Nevertheless, further studies are necessary to elucidate these conflicting findings.

**Table 5:** Peripheral-blood-mononuclear-cells-based biomarkers for ICIs response in NSCLC, evaluated by flow cytometry.

Biomarker	Key Finding
CCR7^−^ CD45RA^−^	Decreased memory effector CD8^+^ T cells (CCR7^−^ CD45RA^−^) were associated with disease progression [117].
FoxP3^+^ Treg cells	Patients who experienced pseudoprogression had reduced CD4^+^CD25^+^CD127^lo^FoxP3^+^ Treg levels compared to values before treatment with ICIs [113].
Gr-MDSC	Elevated levels of Gr-MDSC are associated with a favorable response to treatment with ICIs [97].
PD-1^+^ CD8 T cells	The response of PD-1^+^ CD8 T cells in patients with disease progression was either absent or occurred late [110].
PD-L1^+^ CD14^+^ monocyte	There was a positive correlation between the percentage of the PD-L1^+^ CD14^+^ monocyte subset and OS [112].
TIGIT^+^ T cells	Increased TIGIT^+^ T cells were associated with disease progression [117].
TIM-3^+^ T cells	The increased frequency of TIM-3^+^ T lymphocytes, either CD4^+^ or CD8^+^ T cells negatively correlates to PFS [114].
TMR	Patients with a TMR ≥ 0.39 had significantly longer median progression-free survival [109].
Treg cells	High frequencies of circulating Treg cells one week after anti-PD-1 therapy were correlated with longer PFS and OS [118].

FoxP3^+^—forkhead box P3; Treg—regulatory T cell; ICI—immune checkpoint inhibitor; Gr-MDSC—granulocytic myeloid-derived suppressor cell; PD-1—programmed cell death protein 1; PD-L1—programmed cell death protein ligand 1; OS—overall survival; TIGIT—T cell immunoglobulin and ITIM domain; TIM-3—T cell immunoglobulin- and mucin-domain-containing 3; PFS—progression-free curvival; TMR—the ratio of Tregs to Lox-1^+^ PMN-MDSCs.

### 3.6. Tumor-Infiltrating Immune Cells

The TME comprises non-malignant stromal cells, bone-marrow-derived cells, and tumor-infiltrating lymphocytes (TILs) [119]. In resume, the TME immune population of consists of macrophages, dendritic cells, natural killer cells, B cells, effector T helper cells, and Treg and cytotoxic T cells [120].

Evidence suggests that PD-L1-positive NSCLC patients treated with ICIs who have stromal CD8^+^ effector T cells as the most abundant TIL subpopulation experience better outcomes (Table 6). However, TILs are distributed heterogeneously, and their predictive value may be diminished in patients expressing multiple markers of T cell exhaustion [121], limiting their association with treatment outcomes.

The measurement of TIL populations using computational methods also shows promise, as the spatial distribution of TILs may be linked to ICI response [122,123]. Studies have classified cohorts into three main phenotypes based on TME inflammation: immune-inflamed, immune-excluded, and immune-deserted. Among these, immune-inflamed phenotypes exhibit better responses to ICIs. Compared to other biomarkers, TIL levels can be more predictive than TMB load for PD-L1-negative patients [124].

Proteins have also been investigated as potential biomarkers for treatment response. Examples include CD24, which co-stimulates clonal expansion of CD4 T-cells [125,126]; CD73, which is involved in lymphocyte differentiation [127,128]; and CD137, which is associated with cancer immunity [129,130]. However, CD24 positivity has been correlated with worse PFS in PD-L1 < 50% patients treated with ICIs based on IHC analyses [125]. In addition, the increase in CD73 and CD137 correlates with better PFS [129], regardless of PD-L1 status [127], but these results are still controversial [131]. The major limitation of this methodology is the scarcity of NSCLC tumor tissue [52].

**Table 6:** Tumor-infiltrating-immune-cells-based biomarkers for ICIs response in NSCLC.

Biomarker	Method	Key Finding
CD8^+^ effector in TILs	IHC	The abundance of stromal CD8^+^ effector T cells within the TILs subpopulation was associated with an improved response to ICIs [121].
TIL distribution	IHC	The spatial arrangement of TILs may be linked to the response to ICIs [123].
TIL distribution	Whole-slide images	The spatial arrangement of TILs may be linked to the response to ICIs [122].
TILs levels	Machine learning	Immune-inflamed phenotypes show better responses to ICIs [124].

TILs—tumor-infiltrating lymphocytes; IHC—immunohistochemistry; ICI—immune checkpoint inhibitor.

### 3.7. Extracellular Vesicles

Extracellular vesicles (EVs), which includes exosomes and microvesicles, play a crucial role in the cellular communication mechanism by transporting bioactive molecules that can influence the extracellular environment and the immune system [132]. EVs derived from tumor tissue have the potential to serve as non-invasive biomarkers due to their molecular composition, which reflects the complexity and heterogeneity of the tumor microenvironment (Table 7) [133]. Some molecules carried by EVs, such as PD-L1, TGF-β1, FasL, TRAIL, COX2, CD39/CD73, CTLA4, and NKG2D, are involved in tumor evasion and immunosuppression, and therefore hold promise as predictive biomarkers for immunotherapy response [134].

Studies have shown that responders to ICIs exhibit higher levels of tetraspanin co-stimulatory molecules, specifically CD9, CD81, and CD63, in EVs. These findings suggest that these molecules may serve as promising biomarkers associated with a better objective response rate (ORR) [135]. Conversely, another study identified the overexpression of TGF-β in EVs as a predictor of poorer outcomes and non-response to ICI treatment [136].

EVs can carry small molecules, such as microRNAs (miRNAs), that are widely studied and can also present predictive value [137]. For instance, EV-miR-625-5p has been described as an independent biomarker of response to ICIs in NSCLC patients with PD-L1 expression ≥50% [138]. Furthermore, pre-treatment concentrations of EVs with an endothelial phenotype (CD41a^−^/CD31^+^/CD45^−^) in the blood have been correlated with longer OS, PFS, and clinical response to ICIs. Proteomic analysis has revealed that responders to ICIs have distinct protein loading in EVs at baseline and during treatment [139].

In terms of conventional biomarkers for immune response, EVs offer an alternative method for measuring PD-L1 levels [140,141]. Studies show that increased exosomal PD-L1 expression is associated with better ORR, OS, and treatment efficacy [140]. However, elevated levels of extracellular vesicles are more common in non-responders [139,141], although they may not always predict a sustained response or survival [141]. A novel biochip has been proposed to quantify PD-1/PD-L1 proteins on the surface of extracellular vesicles and EV PD-1/PD-L1 mRNA (Au SERP). This tool has shown 72.2% accuracy in detecting ICI responders and non-responders [142].

The characterization of reference molecules, including mRNA, lncRNA, miRNA, and proteins, within EVs holds promising potential for identifying novel non-invasive biomarkers associated with immunotherapy response.

**Table 7:** Extracellular-vesicles-based biomarkers for ICIs response in NSCLC.

Biomarker	Method	Key Finding
CD41a^−^/CD31^+^ /CD45^−^ EVs	LC-MS/MS	Pre-treatment concentration of EVs was correlated with survival and ICI response [139].
EV-miR-625-5p	Nanostring nCounter	In anti-PD-1-treated patients, EV-miR-625-5p was found to discriminate favorable outcomes in PD-L1 expression ≥ 50% [138].
PD-L1 EVs	Immunoblot	EVs with high PD-L1 expression were correlated to worse outcomes with ICIs and decreased OS and PFS [141]. Conversely, ICI responders presented a decrease in PD-L1 EVs [140].
Protein EVs and mRNA EVs	AuSERP biochip	Dual single-EV PD-1/PD-L1 mRNA detection identified ICI-responders and non-responders with an accuracy of 72.2% [142].
Tetraspanins EVs (CD9, CD81, CD63)	Flow citometry	ICI responders present higher levels of circulating tetraspanins, CD81, CD9, CD63, and CD81-EVs were significantly associated to a better PFS [135].
TGF-β EVs	ELISA	High expression of TGF-β in EVs is associated with non-responders to ICI and present poorer OS and PFS [136].

LC-MS/MS—liquid chromatography tandem mass spectrometry; EVs—extracellular vesicles; ICI—immune checkpoint inhibitor; PD-1—programmed cell death protein 1; PD-L1—programmed cell death protein ligand 1; OS—overall survival; PFS—progression-free survival; ELISA—enzyme-linked immuno sorbent assay.

### 3.8. Imaging Biomarkers

Medical imaging techniques, such as positron emission tomography-computed tomography (PET-CT), can provide insights into the cellular and molecular properties of tumors. These scans have been used to correlate the expression of PD-1/PD-L1 and the response to treatment (Table 8) [143,144,145,146]. Quantifications occur by detecting ^89^Zr probes that are linked to monoclonal antibodies (mAbs) administered during immunotherapy. Studies utilizing ^89^Zr-nivolumab [145] and ^89^Zr-atezolizumab [143] particles have demonstrated their effectiveness in predicting the response to ICIs. On the other hand, ^89^Zr-durvalumab [146], although considered safe and feasible, only exhibited a weak correlation with treatment response.

The application of positron emission tomography-computed tomography using ^18^F-fluorodeoxyglucose (^18^F-FDG PET-CT) has shown promise in determining the metabolic tumor volume (tMTV), which serves as a reliable predictor of pembrolizumab efficacy. Furthermore, tMTV may be a valuable tool for guiding treatment decisions in patients who require more aggressive therapeutic interventions, such as chemoimmunotherapy [144]. The advantage of imaging biomarkers lies in their non-invasive nature; however, they do not always provide consistent predictive information [146], which requires further investigation.

### 3.9. Microbiome

The respiratory tract microbiome has demonstrated its potential to predict the response to ICI in NSCLC patients [147,148,149]. A dysbiotic signature in the respiratory tract microbiome has been associated with tumor progression and a poorer prognosis (Table 9) [150]. Conversely, a more diverse lung microbiome is linked to higher levels of CXCL9, a chemokine associated with better ICI response [149].

Moreover, the application of 16S RNA sequencing has identified specific microbial enrichments in NSCLC patients with different ICI responses. Poor responders to ICIs showed an enrichment of *Fusobacterium nucleatum* [147], *Haemophilus influenzae*, and *Neisseria perflava* [148]. In contrast, patients with an enrichment of *Veillonella dispar* [148] and *Akkermansia muciniphila (AKK)* [151] demonstrated a favorable response to ICIs.

The use of antibiotics can alter the gut microbiome and is correlated with unfavorable clinical outcomes, as patients treated with antibiotics exhibit lower alpha diversity [152]. Evidence suggests that antibiotic treatment reduces the presence of CXCL9 in the lung tumor microenvironment, which may be associated with lower sensitivity to ICIs [149]. Further studies are needed to fully understand the intricate interactions within the cancer–microbiome–immune axis.

## 4. Conclusions

Precision medicine represents the future direction of clinical cancer management, offering more targeted approaches than traditional systemic treatments. Immunotherapy and targeted therapy have emerged as promising alternatives for lung cancer treatment, particularly in the form of ICIs. While ICIs have shown efficacy in NSCLC through clinical trials, the existing PD-L1 biomarker has limitations in reliably predicting response to ICI therapy due to the heterogeneity of NSCLC cases. This review aims to provide an overview of potential biomarkers currently under development or validation, with the goal of enhancing the categorization of NSCLC patients as either responders or non-responders to ICI therapy in the near future.

## Figures and Tables

**Table 3:** Tumor-mutation-burden-based biomarkers for ICIs response in NSCLC.

Biomarker	Method	Key Finding
TMB	cfDNA	Patients with baseline TMB > 16/Mb displaying longer OS in a long-term follow-up after ICI treatment [82].
TMB	Genomic sequencing	ICI-treated patients with TMB > 10/Mb exhibit increased PFS [85]. ICI-treated patients with higher TMB rates present higher PFS [86]. Atezolizumab-treated patients with TMB > 13.6/Mb showed improved PFS as compared to chemotherapy-treated patients [84].

TMB—tumor mutation burden; cfDNA—cell free DNA; OS—overall survival; ICI—immune checkpoint inhibitor; PFS—progression free survival; bTMB—blood-based tumor mutation burden.

**Table 8:** Imaging biomarkers for ICIs response in NSCLC, evaluated by PET-CT.

Biomarker	Key Finding
tMTV (^18^F-FDG PET_CT)	tMTV ≥ 75cm^3^ can indicate unfavorable outcomes in aNSCLC treated with pembrolizumab [144].
^89^Zr-atezolizumab	Higher pre-treatment signal of ^89^Zr-atezolizumab in atezolizumab responders [143].
^89^Zr-durvalumab	Higher pre-treatment signal of ^89^Zr-durvalumab in durvalumab responders [146].

tMTV—metabolic tumor volume; ^18^F-FDG—^18^F-fluorodeoxyglucose; PET-CT—positron emission tomography-computed tomography; aNSCLC—advanced non-small cell lung cancer.

**Table 9:** Microbiome-based biomarkers for ICIs response in NSCLC.

Biomarker	Method	Key Finding
*Akkermansia* *Muciniphila (AKK)*	Metagenomic sequencing	Patients with partial response or stable disease during treatment with ICIs exhibit higher levels of *AKK* [151].
Lung microbiome	Genomic sequencing	Patients with a dysbiotic signature had tumor progression and worse prognosis [150]. Patients who experienced disease progression during treatment with ICIs have a reduced diversity of the lung microbiome [149].

ICI—immune checkpoint inhibitor; *AKK—Akkermansia muciniphila*.

## Data Availability

No new data were created.
